# Nitrogen Limited Red and Green Leaf Lettuce Accumulate Flavonoid Glycosides, Caffeic Acid Derivatives, and Sucrose while Losing Chlorophylls, Β-Carotene and Xanthophylls

**DOI:** 10.1371/journal.pone.0142867

**Published:** 2015-11-16

**Authors:** Christine Becker, Branimir Urlić, Maja Jukić Špika, Hans-Peter Kläring, Angelika Krumbein, Susanne Baldermann, Smiljana Goreta Ban, Slavko Perica, Dietmar Schwarz

**Affiliations:** 1 Department of Modelling and Knowledge Transfer, Leibniz Institute of Vegetable and Ornamental Crops e.V., Grossbeeren, Germany; 2 Department of Plant Sciences, Institute for Adriatic Crops and Karst Reclamation, Split, Croatia; 3 Department of Applied Sciences, Institute for Adriatic Crops and Karst Reclamation, Split, Croatia; 4 Department of Plant Quality, Leibniz Institute of Vegetable and Ornamental Crops e.V., Grossbeeren, Germany; 5 Department of Agriculture and Nutrition, Institute of Agriculture and Tourism, Poreč, Croatia; 6 Department of Plant Nutrition, Leibniz Institute of Vegetable and Ornamental Crops e.V., Grossbeeren, Germany; Universidade Federal de Vicosa, BRAZIL

## Abstract

Reduction of nitrogen application in crop production is desirable for ecological and health-related reasons. Interestingly, nitrogen deficiency can lead to enhanced concentrations of polyphenols in plants. The reason for this is still under discussion. The plants’ response to low nitrogen concentration can interact with other factors, for example radiation intensity. We cultivated red and green leaf lettuce hydroponically in a Mediterranean greenhouse, supplying three different levels of nitrogen (12 mM, 3 mM, 0.75 mM), either in full or reduced (-50%) radiation intensity. In both red and green lettuce, we found clear effects of the nitrogen treatments on growth characteristics, phenolic and photosynthetic compounds, nitrogen, nitrate and carbon concentration of the plants. Interestingly, the concentrations of all main flavonoid glycosides, caffeic acid derivatives, and sucrose increased with decreasing nitrogen concentration, whereas those of chlorophylls, β-carotene, neoxanthin, lactucaxanthin, *all trans*- and *cis*-violaxanthin decreased. The constitutive concentrations of polyphenols were lower in the green cultivar, but their relative increase was more pronounced than in the red cultivar. The constitutive concentrations of chlorophylls, β-carotene, neoxanthin, *all trans*- and *cis*-violaxanthin were similar in red and green lettuce and with decreasing nitrogen concentration they declined to a similar extent in both cultivars. We only detected little influence of the radiation treatments, e.g. on anthocyanin concentration, and hardly any interaction between radiation and nitrogen concentration. Our results imply a greater physiological plasticity of green compared to the red lettuce regarding its phenolic compounds. They support the photoprotection theory regarding anthocyanins as well as the theory that the deamination activity of phenylalanine ammonia-lyase drives phenylpropanoid synthesis.

## Introduction

Nitrogen (N) is a macronutrient essential for optimal plant growth and development. It is involved in many metabolic processes, for instance in amino acid and protein biosynthesis [1]. Nitrogen is massively used as fertilizer in crop production to ensure high yields. Unfortunately, these fertilizers are likely to leach from the crop production system into the environment where they can cause a multitude of problems. According to Galloway et al. [2], high levels of nitrate in the groundwater are a serious health risk, especially for infants, while ecosystems that were originally poor in nitrogen can be suffering from a dramatic loss of species due to nitrogen influx. Therefore, a reduction of nitrogen application in crop production is desirable.

Nitrogen supply can be used as a tool to manipulate plant quality: Low nitrogen availability increases for example the concentration of phenolic compounds in plants [3]. Main phenolic compounds in red leaf lettuce (*Lactuca sativa* L.) are caffeic acid derivatives (chicoric acid, chlorogenic acid and caffeoylmalic acid) as well as red and colorless flavonoid (cyanidin, quercetin and luteolin) glycosides [4]. The cyanidin glycoside, an anthocyanin, absorbs photons from the yellow-green wavebands and is, thus, responsible for the color of the red leaves [5]. The phenolic profile of green leaf lettuce is very similar to that of the red cultivar, except that they do not synthesize anthocyanins and contain overall lower concentrations of the other compounds.

Epidemiological studies indicate a strong link between a diet rich in polyphenols and lower incidence of cardiovascular disease and some kinds of cancer, for instance prostate cancer [6]. For many of the above mentioned phenolic compounds, health promoting effects have been demonstrated *in vitro* [7]. Yet, administering single substances as dietary supplement bears the risk of overdosing with negative effects on human health [8]. Furthermore, synergistic and additive effects of dietary polyphenols as well as their food matrix are considered meaningful [6]. Hence, it appears wise to generally enhance their concentration in crops.

Although plants commonly respond to low nitrogen concentration with the accumulation of phenolic compounds, there have been a few contrasting reports [3]. To our knowledge, the only existing study on the response of lettuce phenolics to nitrogen concentration assessed only the sum of total water soluble plant phenolics [9] instead of gathering detailed data on flavonoid glycosides and caffeic acid derivatives. It is well possible that these two groups respond differently to abiotic influences as shown by previous studies [4,10]. The mechanism behind the response of polyphenols to nitrogen deficiency is still controversial:

1. The C/N ratio-theory: The accumulation might simply be driven by the carbon/nitrogen ratio in the plant, resulting in a general shift from nitrogen- towards carbon-based metabolites under N deficiency [11]. Flavonoid glycosides and caffeic acid derivatives do not contain nitrogen.
2. The deamination-theory: Both groups of phenolic compounds are synthesized via the phenylpropanoid pathway in plants, whose first enzyme, phenylalanine ammonia-lyase (PAL), is the link between primary and secondary metabolism [3]. It releases ammonia from phenylalanine, thereby increasing the level of available nitrogen in the plant and of precursors for phenylpropanoid biosynthesis [12]. The expression of PAL genes increases under nitrogen depletion [13,14].
3. The photoprotection-theory: Flavonoid glycosides and caffeic acid derivatives absorb radiation and scavenge reactive oxygen species (ROS). ROS are produced permanently by the photosynthetic apparatus but especially under biotic and abiotic stress, such as nutrient deficiency [15]. During N deficiency, chlorophyll concentrations and photosynthesis decrease [16]. A malfunctioning photosystem renders the plant in danger of oxidative damage which can explain the increasing concentrations of flavonoids [17].

The antioxidant activity of quercetin, luteolin and cyanidin glycosides and as well as caffeic acid derivatives is equal to or even outperforms ascorbic acid’s and ɑ-tocopherol’s [18]. Extracted from lettuce, they can absorb oxygen radicals [19].

Anthocyanins can shield the photosynthetic apparatus from excess radiation and, hence, may be synthesized to prevent photo-oxidative damage [20], leaving green lettuce potentially more vulnerable to radiation under nitrogen deficiency than red cultivars.

With and without nitrogen deficiency flavonoid biosynthesis in *Arabidopsis thaliana* increases with photosynthetically active radiation—especially regarding anthocyanins [21]. The positive relationship between photosynthetically active radiation and flavonoid glycosides in lettuce has also been demonstrated [22]. In tomato leaves, nitrogen deficiency and radiation have been found to interact strongly in their effects on flavonoids and caffeic acid derivatives [14].

The photosynthetic rate in nitrogen-deficient lettuce is lowered [16] and hence the plant’s carbohydrate pool is reduced. Sugar concentration has been related to flavonoid biosynthesis in several plants species and organs [23–25].

Among the carotenoids found in lettuce are β-carotene, lutein, neoxanthin, lactucaxanthin and violaxanthin [26] which are lipid soluble tetraterpenoids; in plants they are mainly found in plastids [27]. Lutein, β-carotene, neoxanthin, lactucaxanthin and violaxanthin are major xanthophyll pigments of the light harvesting complex of lettuce photosystem II and light harvesting antenna [28–30]. Apart from being accessory pigments, they are involved in non-photochemical quenching (dissipating excess energy from the photosystems as heat) and are also potent ROS scavengers [31,32]. Taking their various functions into account, their concentrations might decrease in response to nitrogen deficiency because they are part of a photosystem that is being down-sized due to lack of chlorophyll. Alternatively, their concentration might increase due to the enhanced formation of reactive oxygen species that has been observed in mineral deficient plant roots [33]. A field experiment with soil grown butterhead lettuce found lower total carotene concentrations in leaves with decreased application of nitrogen fertilizer [34]. However, their low N treatment was additionally lacking phosphorous, sulphur and potassium so the effects cannot clearly be attributed to N limitation. Furthermore, more recently, N limitation was listed as a factor which leads to accumulation of β-carotene [31]. To our knowledge, the detailed effect of nutrient limitation on single xanthophylls in leaf lettuce has received little attention so far. Coria-Cayupán et al. [35] did study lettuce chlorophylls and carotenoids in detail but investigated the effect of different organic fertilizers that differed in a multitude of other aspects, for example heavy metals and pH.

In the experiment presented here, we studied the effects of nitrogen concentration and radiation intensity on the concentration of flavonoid glycosides and caffeic acid derivatives in red and green leaf lettuce as well as their chlorophyll and carotenoid concentrations, paying close attention to possibly occurring interactions. Red and green lettuce genotypes were chosen due to their different phenolic profiles, mainly regarding anthocyanins. We studied the long term effects in a typical Mediterranean greenhouse to obtain results of practical relevance and obtained data on the secondary metabolites in great detail via HPLC-DAD-ESI-MS² and UHPLC-DAD-APCI-TOF.

We investigated the following hypotheses: In red and green lettuce, nitrogen deficiency results in (1) increased concentrations of flavonoid glycosides and of caffeic acid derivatives as well as (2) decreased concentrations of chlorophylls and carotenoids. (3) Green lettuce displays a stronger response due to its lower level of photoprotection. (4) The concentration of sugar correlates to the concentration of phenolics in lettuce. (5) Nitrogen deficiency and radiation intensity interact in their effects on the studied characteristics.

In addition, we discussed our results in the context of proposed theories on the underlying mechanisms of how N deficiency affects plant phenolics.

## Material and Methods

### Plant Cultivation

The experiment was conducted in Split, Croatia (43°31'N, 16°27' E), from 28 January till 16 April, 2013, in a glass covered greenhouse of the Institute for Adriatic Crops and Karst Reclamation which was part of their facilities. No specific permission was required. No endangered or protected species were involved. The mean temperature in the greenhouse was 17.6°C (min: 4°C, max: 30°C). Red and green Lollo lettuce (*Lactuca sativa* L. var. *crispa* L., cv. Satine and cv. Lugano, RijkZwaan, De Lier, The Netherlands) was sown in rockwool cubes, kept at 10°C for two days for germination and subsequently grown in a conventional greenhouse until the experiment started. Before the experiment, all plants received the standard nutrient solution which contained 12 mM nitrogen (see below for the recipe). Four weeks after sowing when plants had developed four true leaves, they were transferred into the experimental setting where they were grown hydroponically, using nutrient film technique, with 20 cm distance between plants. The nutrient solution was prepared carefully with the following composition per liter: 1 mM H_2_PO_4_, 6 mM K^+^, 7 mM Ca^2+^, 1.5 mM Mg^2+^, 2 mM SO_4_^2-^ and microelements: Fe^2+^ 35 μM, Mn^2+^ 10 μM, B 20 μM, Cu^2+^ 0.5 μM, MoO_4_^2-^ 0.5 μM and 4 μM Zn^2+^. Three stock solutions were prepared to obtain nutrient solution containing three different concentrations of nitrogen: 0.75 (low), 3 (medium), and 12 mM (high), 95% of which was in the form of nitrate. The nutrient solutions had EC values of 2.3 ± 0.1 dS m^−1^, pH values of 6.3 ± 0.2 (adjusted by H_2_SO_4_) and were renewed weekly. PH and EC values were controlled daily and adjusted if necessary.

The greenhouse area was divided into four blocks, two of which were covered with a shading net, reducing the photosynthetic photon flux density (PPFD) by 50%. As the greenhouse cover already removed 30% of the PPFD, plants under the net only received 35% of the outside PPFD. Each block contained three gullies, one for each nitrogen concentration-nutrient solution, holding both cultivars. This way, we obtained three replicates for each cultivar, radiation intensity, and nitrogen concentration. The greenhouse was not heated and we followed the natural light cycle, no additional lighting was supplied. Data on radiation intensity was obtained from the Croatian Meteorological and Hydrological Service, Department of Meteorological Research, station Split. Inside the greenhouse, the mean PPFD during the day was 678 μmol m^−2^ s^−1^ during the last week before harvest, with a mean daily maximum of 1330 μmol m^−2^ s^−1^. The diurnal variation is depicted in S1 Fig. Previous experimental data showed that the days directly before harvest are most influential on the flavonoid glycoside concentration in lettuce [4]. Greenhouse glass has a good transmissibility for visible and near infrared wavebands but only allows a low percentage of UVA radiation to pass.

Aboveground organs of all plants were harvested when plants from the high and medium nitrogen-treatments had formed marketable heads. The harvest took place 50 days after planting.

### Plant growth characteristics

At the harvest date, the mean head weight of five plants per cultivar, radiation intensity, nitrogen-concentration, and replicate were assessed. Furthermore, we counted the number of leaves (minimum length: 1.5 cm) of three lettuce heads for each cultivar, radiation intensity, nitrogen concentration, and replicate.

### Sample Preparation

A mixed sample from three plants was prepared for each cultivar, radiation intensity, nitrogen-concentration, and replicate. Only limp or deteriorated outer leaves were removed. Within 30 min after harvesting, the plants were cut in smaller pieces, mixed and frozen at -70°C until lyophilized (FreeZone 2.5, Labconco, Kansas City, USA), and ground with an ultracentrifuge mill (hole size: 0.25 mm; ZM 200, Retsch, Haan, Germany).

### Analyses of flavonoid glycosides and caffeic acid derivatives

Flavonol and flavone glycosides as well as caffeic acid derivatives were analyzed via HPLC-DAD-ESI-MS² according to Becker et al. [4]. In short, 0.5 g of lyophilized, pulverized lettuce powder was extracted with 25 ml of aqueous methanol (50% MeOH) for 90 min at room temperature. The extract was centrifuged, the supernatant cleaned with PTFE-filters, and analyzed via HPLC-DAD-ESI-MS^²^. The anthocyanin method was similar, except for a slightly different composition of the extraction agent and a shorter extraction time: The extraction agent was acidified aquaeous methanol (40% MeOH, 10% acetic acid). Extraction of anthocyanin glycosides took 15 min. The system used for analysis consists of an Agilent HPLC series 1100 Ion Trap (Agilent, Waldbronn, Germany). The compounds were separated on a Prodigy column (ODS 3, 150 x 3 mm, 5 μm, 100 Å; Phenomenex, Aschaffenburg, Germany) with a security guard C18 (ODS 3, 4 x 3 mm, 5 μm, 100 Å) at 30°C using a water/acetonitrile gradient. DAD wavelength for quantification was 330 nm for caffeic acid derivatives, 350 nm for flavonol and flavone glycosides, and 520 nm for anthocyanidin glycosides.

Based on the measured concentrations of flavonoids and caffeic acid derivatives, we calculated the amount of nitrogen in the plant that can be attributed to the enzymatic activity of PAL. For each molecule of quercetin, luteolin, cyanidin and chlorogenic acid, one molecule of phenylalanine is de-aminated, rendering one atom of nitrogen available once again for plant metabolism. As chicoric acid contains two caffeic acid moieties, two phenylalanine molecules are metabolized. Based on their molecular mass, we elucidated how many molecules of each flavonoid and caffeic acid derivative were present in the plant, on the basis of their molecular mass. This way, we calculated which percentage of plant nitrogen may be attributable to the enzymatic activity of PAL. We used this data to estimate the quantitative relevance of the PAL-attributable share of total N.

### Analyses of chlorophyll a and b, β-carotene, lutein, neoxanthin, lactucaxanthin and violaxanthin

From the freeze-dried material, 5–10 mg were weighed in an Eppendorf tube, 500 μl of MeOH/THF (1:1,v/v)-solution was added, the suspension was shaken for 5 min at 1400 rpm and 20°C and afterwards centrifuged for 5 min at 4500 rmp at 20°C. The supernatant was collected in a 4 ml glass vial and the pellet was extracted again twice. In a nitrogen stream, the solvent was evaporated and resolved in 20 μl of dichlormethane and 180 μl iso-propanol. This mixture was filtered through a syringe filter (PTFE 0.2 μm) before being analyzed by the UHPLC. Injection volume was 5 μl. The carotenoids were separated on a C30-column (YMC Co. Ltd Japan, YMC C30, 100 x 2.1 mm, 3 μm) and analyzed on an Agilent Technologies 1290 Infinity UHPLC coupled with an Agilent Technologies 6230 TOF LC/MS equipped with an APCI ion source. The gas temperature was set to 325 C at a flow rate of 8 l min^−1^, the vaporizer to 350°C and the nebulizer pressure was set to 3 psi. The voltage was set to 3500 V and a fragmentor voltage of 175 V was applied at a corona current of 6.5 μA. Mixtures of methanol, methyl-tert-butyl-ether and water in different volume ratios (solvent A: 81/15/4 and solvent B: 6/90/4) were used as the mobile phases at a flow rate of 0.2 ml min^−1^. The positive ionization was enhanced by addition of 20 mM ammonium acetate. The carotenoids were separated in gradient mode from 100% (10 min isocratic) to 0% solvent A within 30 min. Stock solutions of the authentic standards (β-carotene, lutein, neoxanthin, and violaxanthin) were prepared individually and their concentrations determined spectro-photometrically [36]. Identification was achieved by co-chromatography with references substances. External standard calibration curves were used for quantification by dose-response curves. Lactucaxanthin was calculated in lutein equivalents.

Neoxanthin and *all trans*-violaxanthin were identified by comparison to standard substances. The peak identified as *cis*-violaxanthin displayed the same molecular mass as violaxanthin while showing a characteristic additional peak in its spectrum indicating one *cis*-bond. Under our conditions, the mass spectrometric fragments also indicated that it is a violaxanthin isomer and not neoxanthin as water is cleaved more easily from the latter. Hence, the [M–H_2_O]^+^ signal is more abundant in a neoxanthin peak compared to a violaxanthin peak as confirmed by the authentic standards under our analysis conditions.

### Analyses of nitrogen, nitrate and carbon concentration

Total N and C were determined after dry oxidation by the Dumas method (Elementar Vario EL, Hanau, Germany). The content of nitrate ions (NO_3_^−^) was determined in head samples potentiometrically with Sure-Flow^®^ electrode (Orion-Research, Beverly; USA). Both methods were applied as described in the manufacturer’s manual.

### Analysis of sugar concentration

For sugar analysis, 10 mg of freeze-dried plant material were extracted with 800 μl of aquaeous ethanol (80% EtOH), vortexed and incubated for 20 min at 78°C. After centrifugation at 14000 rpm and 4°C for 10 min, the supernatant was transferred into a new Eppendorf reaction tube. The extraction was repeated twice with 400 μl 50%-EtOH. Of the combined supernatants, 5 μl were and analyzed via enzymatic essay in microplates as described in [37].

### Statistical Analyses

**Fig 1:**
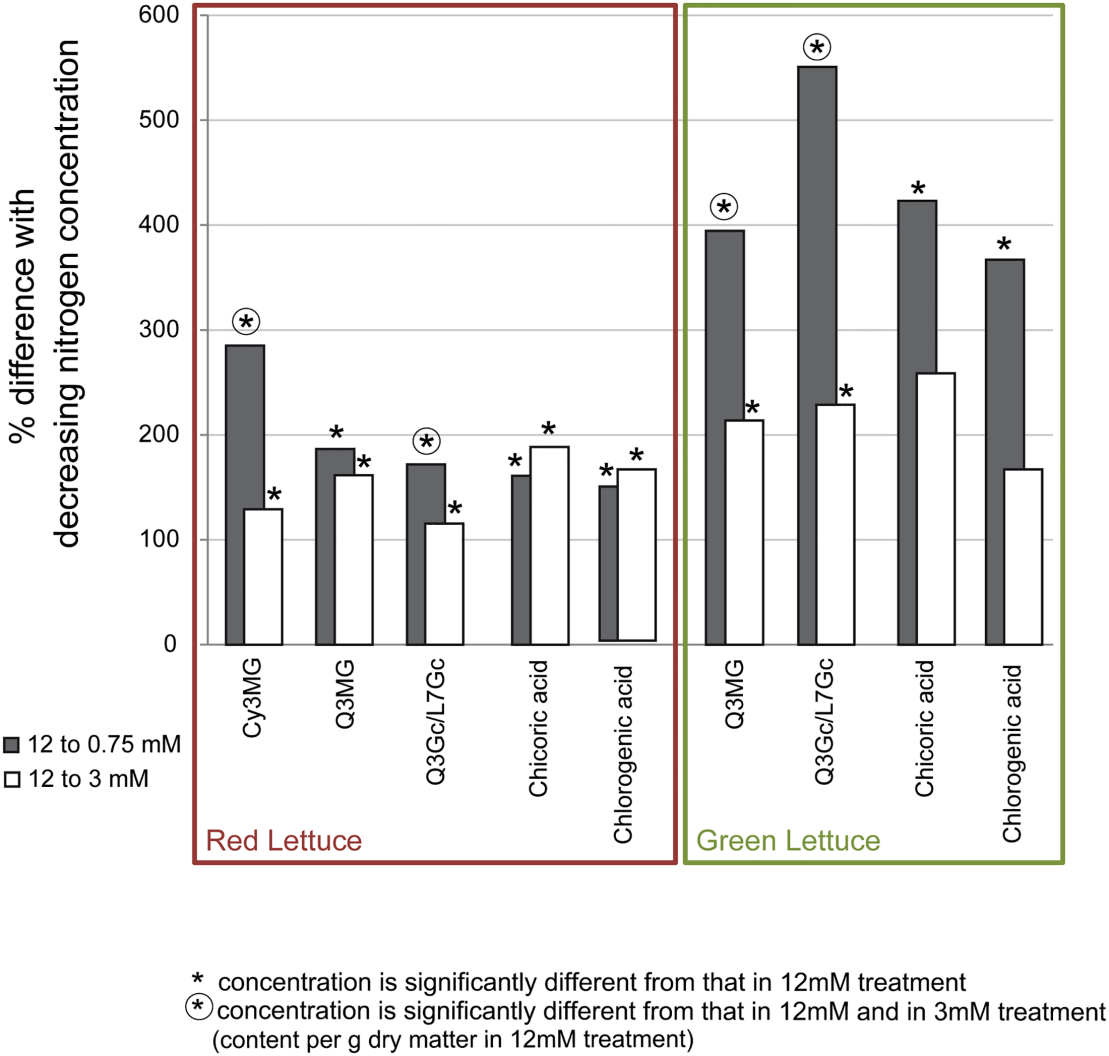
Phenolic compounds’ response to decreased nitrogen concentration. Flavonoid glycosides and caffeic acid derivatives were present in different concentrations in red and green lettuce, cultivated at 12, 3 or 0.75 mM nitrogen in the nutrient solution. The percentage of difference between the 3 and 12 mM treatments is represented by the white bars, difference between the 0.75 and 12 mM treatments is represented by the grey bars. Asterisks indicate that the value is significantly different from that in 12 mM treatment, asterisks in circles indicate 0.75 mM treatment values which were significantly different from those in the 12 and the 3 mM treatments (two-way ANOVA, Tukey HSD test, α = 0.05, n = 3; as calculated based on the concentrations and listed in Table 2). Cy3MG = cyanidin-3-*O*-(6΄΄-*O*-malonyl)-glucoside, Q3MG = quercetin-3-*O*-(6΄΄-*O*-malonyl)-glucoside, Q3Gc/L7Gc = quercetin-3-*O*-glucuronide and luteolin-7-*O*-glucuronide (sum).

**Fig 2:**
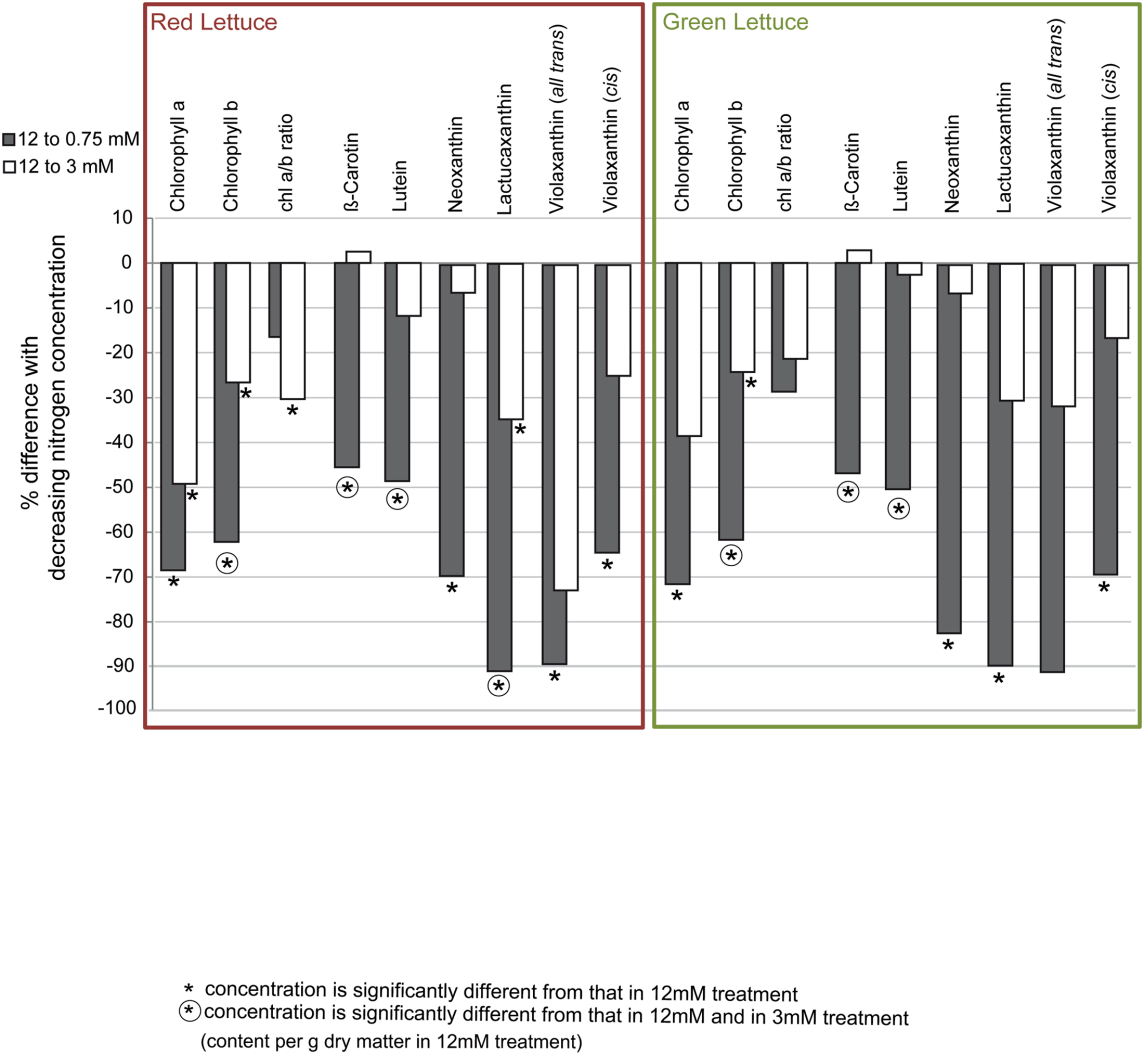
Chlorophylls’ and carotenoids’ response to decreased nitrogen concentration. Chlorophylls and carotenoids were present in different concentrations in red and green lettuce, cultivated at 12, 3 or 0.75 mM nitrogen in the nutrient solution. The percentage of difference between the 3 and 12 mM treatments is represented by the white bars, difference between the 0.75 and 12 mM treatments is represented by the grey bars. Asterisks indicate that the value is significantly different from that in 12 mM treatment, asterisks in circles indicate 0.75 mM treatment values which were significantly different from those in the 12 and the 3 mM treatments (two-way ANOVA, Tukey HSD test, α = 0.05, n = 3; as calculated based on the concentrations and listed in Table 3).

In order to study the effect of radiation intensity and nitrogen concentration of the nutrient solution on the concentration of several secondary metabolites in red and green leaf lettuce and to detect possible interaction between them, factorial ANOVA was performed. Because the 3-factorial ANOVA (Fisher’s F-test; factor 1: nitrogen concentration, factor 2: radiation intensity, factor 3: cultivar) detected significant interactions between nitrogen concentration and cultivar, 2-factorial ANOVA was conducted for green and red lettuce separately (Fisher’s F-test; factor 1: nitrogen concentration, factor 2: radiation intensity), followed by Tukey’s Honest Significant Difference (HSD) test. Kolmogorov-Smirnov test for normal distribution of the residuals was performed and did not oppose the evaluation by factorial ANOVA. For Figs 1 and 2, relative differences between the treatments were calculated based on the respective concentrations of phenolics, chlorophylls, and carotenoids we measured in our analyses. Correlation analysis was performed on the concentrations of sugars and of phenolics. A significance level of α = 0.05 was applied. Calculations were performed using STATISTICA (version 10, Statsoft Inc., Tulsa, USA) and Microsoft Excel 2010.

## Results and Discussion

### Red and green lettuce: genotypic difference

The results of the evaluation via 3-factorial ANOVA (S1–S3 Tables) showed that red and green lettuce differed significantly regarding all measured phenolic compounds (S1 Table) as well as chlorophyll b, β-carotene, lutein, neoxanthin and *cis*-violaxanthin (S2 Table). Their concentrations were higher in red than in green lettuce. Compared to the green, red lettuce contained higher concentrations of total carbon but lower concentration of total nitrogen and nitrate, gained a lower head mass and had a lower number of leaves (S3 Table). This is in line with the “plant defense theory” [38,39], predicting higher allocation costs for red than for green lettuce due to higher concentrations of secondary compounds in the first one, meaning that red lettuce allocated carbon into the biosynthesis of phenolics instead of growth which shows in its lower head mass.

Furthermore, the 3-factorial ANOVA (S1–S3 Tables) showed significant interactions between nitrogen concentration and cultivar regarding chlorogenic and chicoric acid, quercetin-3-*O*-(6”-*O*-malonyl)-glucoside, total nitrogen, nitrate, total carbon, head mass and sucrose. We therefore conducted the following evaluations via 2-factorial ANOVA, for red and green lettuce separately (Tables 1–4).

**Table 1:** Growth characteristics and nitrogen, carbon and nitrate concentration of red and green lettuce cultivated at different nitrogen concentration and photosynthetic photon flux density.

	Nitrogen	PPFD	Head mass	number of leaves	total N	NO_3_	total C	C/N ratio
**Red Lettuce**	0.75	678	18.6	10.3	9.7	0.09	396.9	41.0 a
	3	678	76.0	17.7	16.9	0.05	382.9	22.8 c
	12	678	204.4	23.0	32.9	5.02	388.1	11.8 d
	0.75	339	17.4	11.3	10.9	0.09	395.3	36.4 b
	3	339	70.5	16.7	18.2	0.08	379.9	20.9 c
	12	339	184.2	22.8	33.5	5.37	384.5	11.5 d
**Main effects**								
0.75 mM N			18.0 c	10.8 c	10.3 c	0.09 b	396.1 a	38.7
3 N			73.3 b	17.2 b	17.6 b	0.06 b	381.4 c	21.8
12 N			192.9 a	22.9 a	33.2 a	5.19 a	386.3 b	11.7
678 μmol PPFD			99.7	17.0	19.8 B	1.72	389.3 A	25.2
339 μmol PPFD			90.7	17.5	20.9 A	1.85	386.5 B	22.9
**Significances**								
N			*	*	*	*	*	*
PPFD			ns	ns	*	ns	*	*
N * PPFD			ns	ns	ns	ns	ns	*
**Green Lettuce**	0.75	678	28.7	13.7	9.5	0.04 c	395.4 a	41.6
	3	678	93.6	22.3	18.6	0.46 c	384.0 b	20.8
	12	678	292.3	27.3	37.7	8.58 b	367.1 c	9.8
	0.75	339	26.0	14.3	12.1	0.56 c	396.8 a	33.5
	3	339	99.6	21.7	21.3	0.22 c	368.1 c	17.3
	12	339	278.5	27.7	39.1	10.08 a	362.2 c	9.3
**Main effects**								
0.75 mM N			27.4 b	14.0 c	10.8 c	0.30	396.1	37.5 a
3 mM N			96.6 b	22.0 b	19.9 b	0.34	376.1	19.1 b
12 mM N			285.4 a	27.5 a	38.4 a	9.33	364.6	9.5 c
678 μmol PPFD			138.2	21.1	21.9 B	3.02	382.2	24.1 A
339 μmol PPFD			134.7	21.2	24.1 A	3.62	375.7	20.0 B
**Significances**								
N			*	*	*	*	*	*
PPFD			ns	ns	*	*	*	*
N * PPFD			ns	ns	ns	*	*	ns

Nitrogen (N) concentration in the nutrient solution is given in millimol per liter (mM). Photosynthetic photon flux density (PPFD) is given in μmol m^−2^ s^−1^. Head mass is given in gram fresh matter. Nitrogen, carbon (C) and nitrate (NO_3_) concentration is given in milligram per gram dry matter. Data was evaluated via two-way ANOVA, factors: mM N and PPFD, α = 0,05, followed by Tukey HSD test (mean, n = 3). Identical letters indicate that values do not differ significantly. Asterisks indicate significantly influential factors.

**Table 2:** Concentrations of phenolic compounds, percent of total N invested in chlorophylls and percent of total N attributable to PAL activity in red and green lettuce cultivated at different nitrogen concentration and photosynthetic photon flux density.

	Nitrogen	PPFD	Cy3MG	Q3GC/L7Gc	Q3MG	Chicoric acid	Chlorogenic acid	Total N invested in Chl a+b	Total N attributable to PAL activity
**Red Lettuce**	0.75	678	2.81	2.04	6.17	2.96	1.16	0.55	5.29
	3	678	1.77	1.68	6.18	3.45	1.38	0.61	3.02
	12	678	0.94	0.75	2.14	1.02	0.46	0.52	0.55
	0.75	339	2.27	1.79	5.52	3.02	1.26	0.59	4.43
	3	339	1.25	1.36	4.49	3.16	1.20	0.57	2.31
	12	339	0.38	0.66	1.94	1.27	0.52	0.53	0.53
**Main effects**									
0.75 mM N			2.54 a	1.91 a	5.84 a	2.99 a	1.21 a	0.57	4.86 a
3 mM N			1.51 b	1.52 b	5.33 a	3.30 a	1.29 a	0.59	2.67 b
12 mM N			0.66 c	0.70 c	2.04 b	1.15 b	0.49 b	0.53	0.54 c
678 μmol PPFD			1.84 A	1.49 A	4.83	2.48	1.00	0.56	2.95 A
339 μmol PPFD			1.30 B	1.26 B	3.98	2.48	1.00	0.56	2.42 B
**Significances**									
N			*	*	*	*	*	ns	*
PPFD			*	*	ns	ns	ns	ns	*
N * PPFD			ns	ns	ns	ns	ns	ns	ns
**Green Lettuce**	0.75	678		1.31	2.00	1.34	0.37	0.31	1.94
	3	678		0.71	1.23	0.87	0.23	0.53	0.63
	12	678		0.25	0.50	0.47	0.13	0.31	0.14
	0.75	339		1.15	1.70	1.64	0.47	0.49	1.70
	3	339		0.53	1.11	1.17	0.26	0.44	0.58
	12	339		0.13	0.24	0.10	0.05	0.44	0.05
**Main effects**									
0.75 mM N				1.23 a	1.85 a	1.49 a	0.42 a	0.40	1.82 a
3 mM N				0.62 b	1.17 b	1.02 ab	0.24 ab	0.48	0.60 b
12 mM N				0.19 c	0.37 c	0.29 b	0.09 b	0.38	0.09 b
678 μmol PPFD				0.76	1.25	0.90	0.24	0.38	0.90
339 μmol PPFD				0.60	1.02	0.97	0.26	0.46	0.78
**Significances**									
N				*	*	*	*	ns	*
PPFD				ns	ns	ns	ns	ns	ns
N * PPFD				ns	ns	ns	ns	ns	ns

Nitrogen (N) concentration in the nutrient solution is given in millimol per liter (mM). Photosynthetic photon flux density (PPFD) is given in μmol m^−2^ s^−1^. Phenolics concentration is given in milligram per gram dry matter. Data was evaluated via two-way ANOVA, factors: mM N and PPFD, α = 0.05, followed by Tukey HSD test (mean, n = 3). Identical letters indicate that values do not differ significantly. Asterisks indicate significantly influential factors. Cy3MG = cyanidin-3-O-(6΄΄-O-malonyl)-glucoside, Q3MG = quercetin-3-O-(6΄΄-O-malonyl)-glucoside, Q3Gc/L7Gc = quercetin-3-O-glucuronide and luteolin-7-O-glucuronide.

**Table 3:** Concentrations of carotenoids in red and green lettuce cultivated at different nitrogen concentration and photosynthetic photon flux density.

	Nitrogen	PPFD	Chlorophyll a	Chlorophyll b	β-Carotene	Lutein	Lactucaxanthin	Neoxanthin	*all trans-V*iolaxanthin	*cis-V*iolaxanthin
**Red Lettuce**	0.75	678	0.50	0.36	0.05	0.07	0.002	0.037	0.0002	0.02
	3	678	0.91	0.74	0.10	0.13	0.055	0.117	0.0001	0.04
	12	678	1.74	1.02	0.12	0.16	0.119	0.173	0.0044	0.06
	0.75	339	0.60	0.43	0.06	0.08	0.018	0.063	0.0007	0.02
	3	339	0.87	0.78	0.11	0.15	0.096	0.195	0.0021	0.05
	12	339	1.76	1.07	0.09	0.15	0.114	0.159	0.0035	0.06
**Main effects**										
0.75 mM N			0.55 b	0.39 c	0.06 b	0.08 b	0.010 c	0.05 b	0.0004 b	0.02 b
3 mM N			0.89 b	0.76 b	0.11 a	0.14 a	0.076 b	0.16 a	0.0011 ab	0.04 a
12 mM N			1.75 a	1.04 a	0.10 a	0.15 a	0.116 a	0.17 a	0.0040 a	0.06 a
678 μmol PPFD			1.05	0.71	0.09	0.12	0.059	0.11	0.0015	0.04
339 μmol PPFD			1.08	0.76	0.09	0.13	0.076	0.14	0.0021	0.04
**Significances**										
N			*	*	*	*	*	*	*	*
PPFD			ns	ns	ns	ns	ns	ns	ns	ns
N * PPFD			ns	ns	ns	ns	ns	ns	ns	ns
**Green Lettuce**	0.75	678	0.27	0.21	0.03	0.04	0.001	0.002	0.0001	0.01
	3	678	1.03	0.55	0.08	0.10	0.030	0.088	0.0003	0.03
	12	678	1.16	0.69	0.06	0.10	0.042	0.098	0.0007	0.03
	0.75	339	0.59	0.40	0.05	0.07	0.012	0.036	0.0003	0.02
	3	339	0.84	0.66	0.08	0.11	0.059	0.119	0.0021	0.04
	12	339	1.88	0.90	0.09	0.12	0.087	0.123	0.0028	0.05
**Main effects**										
0.75 mM N			0.43 b	0.30 c	0.04 b	0.06 b	0.007 b	0.02 b	0.0002	0.01 b
3 mM N			0.94 ab	0.60 b	0.08 a	0.11 a	0.045 a	0.10 a	0.0012	0.03 a
12 mM N			1.52 a	0.80 a	0.08 a	0.11 a	0.065 a	0.11 a	0.0017	0.04 a
678 μmol PPFD			0.82	0.48 B	0.06	0.08 B	0.025 B	0.06	0.0004 B	0.02 B
339 μmol PPFD			0.11	0.65 A	0.07	0.10 A	0.053 A	0.09	0.0017 A	0.03 A
**Significances**										
N			*	*	*	*	*	*	ns	*
PPFD			ns	*	ns	*	*	ns	*	*
N * PPFD			ns	ns	ns	ns	ns	ns	ns	ns

Nitrogen (N) concentration in the nutrient solution is given in millimol per liter (mM). Photosynthetic photon flux density (PPFD) is given in μmol m^−2^ s^−1^. Concentrations are given in milligram per gram dry matter. Data was evaluated via two-way ANOVA, factors: mM N and PPFD, α = 0.05, followed by Tukey HSD test (mean, n = 3). Identical letters indicate that values do not differ significantly. Asterisks indicate significantly influential factors.

**Table 4:** Concentrations of sugars in red and green lettuce cultivated at different nitrogen concentration and photosynthetic photon flux density.

	Nitrogen	PPFD	glucose	fructose	sucrose
**Red Lettuce**	0.75	678	24.7	28.5	220.5
	3	678	67.6	95.5	78.6
	12	678	68.0	110.9	31.5
	0.75	339	31.7	33.5	199.2
	3	339	80.8	110.9	51.6
	12	339	79.3	110.9	42.7
**Main effects**					
0.75 mM N			28.2 b	31.0 b	209.8 a
3 mM N			74.2 a	103.2 a	65.1 b
12 mM N			73.6 a	110.9 a	37.1 c
678 μmol PPFD			53.4 B	78.3	110.2
339 μmol PPFD			63.9 A	85.1	97.8
**Significances**					
N			*	*	*
PPFD			*	ns	ns
N * PPFD			ns	ns	ns
**Green Lettuce**	0.75	678	34.6	35.5	184.2
	3	678	74.2	94.7	88.2
	12	678	72.1	99.8	37.8
	0.75	339	27.0	28.5	161.4
	3	339	81.0	96.8	59.5
	12	339	67.7	102.5	47.0
**Main effects**					
0.75 mM N			30.8 b	32.0 b	172.8 a
3 mM N			77.6 a	95.8 a	73.9 b
12 mM N			69.9 a	101.2 a	42.4 b
678 μmol PPFD			60.3	76.7	103.4
339 μmol PPFD			58.6	75.9	89.3
**Significances**					
N			*	*	*
PPFD			ns	ns	ns
N * PPFD			ns	ns	ns

Nitrogen (N) concentration in the nutrient solution is given in millimol per liter (mM). Photosynthetic photon flux density (PPFD) is given in μmol m^−2^ s^−1^. Concentrations are given in milligram per gram dry matter. Data was evaluated via two-way ANOVA, factors: mM N and PPFD, α = 0.05, followed by Tukey HSD test (mean, n = 3). Identical letters indicate that values do not differ significantly. Asterisks indicate significantly influential factors.

### Growth characteristics, nitrogen, nitrate and carbon concentration

Results are listed in Table 1. In both red and green lettuce, nitrogen concentration influenced the plants’ head mass immensely. Plants cultivated at 0.75 mM or 3 mM nitrogen in the nutrient solution had only about 9% or 36% of the head mass and about 49% or 78% of the number of leaves of those plants cultivated at 12 mM.

With higher nitrogen concentration in the nutrient solution, the total N concentration strongly increased in leaves of red and green lettuce. Generally, this is also true for the nitrate concentration but there is an interesting detail here. In contrast to total N, the nitrate concentration did not increase steadily with increasing N concentration in the nutrient solution: Plants cultivated at 0.75 mM or 3 mM only contain about 1.5% (red lettuce) and 3.4% (green lettuce), respectively, of the nitrate concentration found in 12 mM-cultivated plants. The drastic drop of nitrate concentration between plants cultivated at 12 mM and 3 mM is very interesting under nutritional aspects as high nitrate concentrations can be harmful. However, the total nitrate concentration never reached high values. The maximum nitrate concentration analyzed was 583 mg kg^−1^ fresh mass of green lettuce cultivated shaded at 12 mM. This is much lower than the nitrate thresholds for greenhouse lettuce imposed by the European Community at 4500 mg kg^−1^ of fresh mass when grown from 1 October to 31 March and at 3000 mg kg^−1^ from 1 April to 30 September.

On this note, we want to highlight that, while the cultivars do not differ markedly regarding total nitrogen concentration, red lettuce contained much lower nitrate concentrations than green lettuce.

Green lettuce plants had the highest total carbon concentration when cultivated at 0.75 mM N. It decreased with increasing nitrogen concentration. Red lettuce plants also had the highest carbon concentration in the 0.75 mM treatment but the second highest was found in the 12 mM and the lowest in the 3 mM treatment. In both red and green lettuce, the mean carbon concentration was significantly higher in unshaded plants compared to shaded ones, even if the absolute difference was not very large. However, the small magnitude of difference (differences due to N concentrations were larger) indicates that the radiation intensity (mean daytime PPFD was 678 μmol m^−2^ s^−1^) was close to the light saturation level. This assumption is supported by a previous study: In artificial lighting, Romaine lettuce is experiencing mild stress when exposed to a PPFD of 600 μmol m^−2^ s^−1^ continuously for 14 h without gaining more biomass than plants cultivated under 400 μmol m^−2^ s^−1^ [40].

The C/N ratio was highest in plants cultivated at 0.75 mM and decreased significantly with increasing N concentration in the nutrient solution, in both red and green lettuce. Furthermore, the C/N ratio was significantly higher in plants cultivated unshaded compared to plants grown under the shading net.

The only interaction between radiation intensity and nitrogen concentration detected among growth characteristics, N, C, and NO_3_ concentrations were regarding C/N ratio of red lettuce and total C and NO_3_ concentration of green lettuce.

### Quercetin, luteolin and cyanidin glycosides, chlorogenic and chicoric acid

In our HPLC-DAD-ESI-MS^2^ analyses of flavonol, flavone, and anthocyanidin glycosides as well as caffeic acid derivatives in red and green leaf lettuce, we identified two quercetin glycosides, one luteolin glycoside, one cyanidin glycoside, and several caffeic acid derivatives. The main phenolic compound was quercetin-3-*O*-(6΄΄-*O*-malonyl)-glucoside followed by chicoric acid (di-*O*-caffeoyltartaric acid) and cyanidin-3-O-(6΄΄-*O*-malonyl)-glucoside, quercetin-3-*O*-glucuronide and luteolin-7-*O*-glucuronide, and chlorogenic acid (5-*O*-caffeoylquinic acid). Green lettuce did not contain any cyanidin-3-*O*-(6΄΄-*O*-malonyl)-glucoside. These compounds were previously reported for red leaf lettuce [41]. Quercetin-3-*O*-glucuronide and luteolin-7-*O*-glucuronide co-eluted and were quantified as sum. Mass spectrometric data suggested that in average they contributed in equal shares to the peak evaluated via DAD. This is in line with data obtained by DuPont et al. [41].

In general, the concentrations of flavonoid glycosides and caffeic acid derivatives strongly increased with decreasing nitrogen concentration, in both red and green lettuce. Results are displayed in Fig 1 and Table 2.

In red lettuce, the concentration of cyanidin-3-*O*-(6”-*O*-malonyl)-glucoside, quercetin-3-*O*-(6”-*O*-malonyl)-glucoside, and quercetin-3-*O*-glucuronide/luteolin-7-*O*-glucuronide were higher in plants cultivated at 3 mM compared to those plants cultivated at 12 mM nitrogen in the nutrient solution. Regarding cyanidin-3-*O*-(6”-*O*-malonyl)-glucoside and quercetin-3-*O*-glucuronide/luteolin-7-*O*-glucuronide the concentrations were further increased in plants cultivated at 0.75 mM while the other compounds were measured in concentrations comparable to the 3 mM treatment. In green lettuce, both quercetin and luteolin glycosides were present in higher concentrations in plants cultivated at 3 mM compared to 12 mM nitrogen in the nutrient solution and these higher concentrations were further increased in plants grown in the 0.75 mM treatment. In red lettuce, the radiation intensity had a significant influence on the concentration of quercetin-3-*O*-glucuronide/luteolin-7-*O*-glucuronide and of cyanidin-3-*O*-(6”-*O*-malonyl)-glucoside. Unshaded plants contained higher concentration of both the glucuronides (1.49 mg g^−1^) and the cyanidin glycoside (1.84 mg g^−1^) compared to their shaded counterparts (1.26 and 1.30 mg g^−1^ respectively). The quercetin-3-*O*-(6”-*O*-malonyl)-glucoside was not significantly influenced by irradiation. In green lettuce, none of the phenolic compounds were increased in unshaded compared to shaded plants. (See Table 2 for details.)

In red lettuce, chicoric and chlorogenic acid concentrations were significantly higher in plants cultivated at 3 mM than in those cultivated in the 12 mM treatment but they were not further increased in plants cultivated at 0.75 mM N. In green lettuce, only the 0.75 mM-cultivated plants contained significantly higher concentrations than the 12 mM N treatment. Neither chicoric nor chlorogenic acid concentration were significantly influenced by radiation intensity, neither in red nor in green lettuce. No interactions between radiation intensity and nitrogen concentration regarding the concentration of phenolic compounds were detected.

The increase of flavonoid glycosides and caffeic acid derivatives in red and green lettuce with decreasing nitrogen concentration, taking interactions with radiation intensity into account, has not been shown this detailed before but is in line with the results found in other plant species like apple and tomato leaves [3,14]. It is remarkable that both flavonoid glycosides as well as caffeic acid derivatives responded to N limitation. In our previous experiments on the impact of moderate PPFD, only flavonoids, not caffeic acid derivatives, were responsive.

Phenolic compounds in green lettuce generally showed a stronger relative increase than in red lettuce when nitrogen concentration in the nutrient solution decreased (Fig 1): The concentration of quercetin and luteolin glycosides, chlorogenic and chicoric acid was 367–551% higher in plants cultivated at 0.75 mM compared to those cultivated at 12 mM. In red lettuce, the concentrations were only higher by 147–187%. Even the cyanidin glycoside that showed the strongest increase of the phenolic compounds in red lettuce was only 285% higher in plants cultivated at 0.75 mM compared to plants cultivated at 12 mM. This can be interpreted as a higher level of constitutive protection of the red compared to the green lettuce, whereas green lettuce appears to have a higher physiological plasticity (e.g. ability to change its physiology in response to a changing environment) in terms of phenolic compounds.

Interestingly, a similar pattern has been found in another study on green and red basil regarding radiation: Green basil responded much more sensitively to increasing radiation intensity than red basil [42]. The authors attributed the more conservative response of red basil to its constitutively higher concentrations of phenolic compounds, especially anthocyanins. This is mirrored by our results regarding N limitation as the highest concentration of phenolics in green lettuce (0.75 mM treatment) just about reaches the level of the lowest concentration in red lettuce (12 mM treatment; see Table 2).

One might argue that lettuce cultivated at low N concentration has higher concentrations of phenolics because it has a lower physiological plant age (displayed by a lower number of leaves) than the 12 mM treatment. The physiological age has been demonstrated to be influential regarding the concentration of phenolic compounds [22]. However, this is contradicted by our results in several cases: While the difference in leaf number is very large between plants from the 3 mM and the 0.75 mM treatment, the differences in concentration are mostly between the 3 mM and the 12 mM treatments (see Table 2, especially regarding the red cultivar).

In a previous study, all flavonoid glycosides in red leaf lettuce were found to increase with increasing radiation intensity while none of the caffeic acid derivatives did [22]. Results obtained in the experiment presented here are largely in line with that. The only difference is that not all flavonoid glycosides responded to radiation intensity. The explanation might lie in the relatively high radiation intensity plants experience in spring in Croatia. In the earlier experiment, some compounds appeared to approach a saturation level at PPFD higher than the maximum studied there (230 μmol m^−2^ s^−1^). Another experiment still showed different quercetin and luteolin glycoside concentrations between plants grown at 410 and 225 μmol m^−2^ s^−1^[4]. In the experiment presented here, plants experienced an average PPFD of 678 μmol m^−2^ s^−1^ during the day for the last week before harvest, with average daily maxima of 1900 μmol m^−2^ s^−1^. This is very likely to exert stress on lettuce plants [40] and even 50% of it may have been enough to maximize biosynthesis of some flavonoids.

### Chlorophyll a and b, β-carotene, lutein, neoxanthin, lactucaxanthin, *all trans*- and *cis*-violaxanthin

In our UHPLC-DAD-APCI-TOF analyses of chlorophylls and carotenoids in red and green leaf lettuce, we identified chlorophyll a and b, neoxanthin, lactucaxanthin and violaxanthin which have been detected in lettuce previously [26]. The ratio between carotenoids reported in the literature differs from the one we found which might be related to differences in genotypes. To our knowledge, the two violaxanthin isomers (*cis* and *all trans*) have not been measured separately in lettuce before.

In red lettuce, chlorophyll a was on average present in higher concentrations than chlorophyll b and the main carotenoids were lutein, neoxanthin and lactucaxanthin, followed by β-carotene and then violaxanthin (based on the mean values of all treatments). In green lettuce, these compounds were present in the same descending order.

In general, the concentration of chlorophylls and carotenoids decreased with decreasing nitrogen concentration in both red and green lettuce. The response of the chlorophylls and carotenoids was more uniform between the red and green lettuce than the response of the phenolic compounds. Results are displayed in Fig 2 and listed in Table 3.

In both red and green lettuce, chlorophyll a and b decreased with decreasing nitrogen concentration (Fig 2). Chlorophyll a concentration was about 70% lower in plants cultivated at 0.75 mM compared to 12 mM in both cultivars. The concentration in plants cultivated at 3 mM was also 40–50% lower than in the 12 mM treatment, however, this difference was only significant regarding red lettuce. The ratio of chlorophyll a and b was only significantly different between red lettuce plants cultivated at 12 and 3 mM. Our results show that chlorophyll b responded more sensitively to the nitrogen treatments than chlorophyll a. In both red and green lettuce, the concentration was 26 ± 2% (mean ± standard deviation) in the 3 mM treatment and even 62% lower in plants cultivated at 0.75 mM compared to those cultivated at 12 mM N. The stronger responsiveness of chlorophyll b than chlorophyll a to the changing nitrogen concentrations agrees with results by Berges et al. [43]. They reported that nitrogen starvation affected photosystem II greater than photosystem I in several species of microalgae. Photosystem II contains chlorophyll a and b in equal parts while photosystem I contains four times more chlorophyll a than b in their light harvesting antennae [27].

In both red and green lettuce, the concentrations of β-carotene, lutein, neoxanthin and both violaxanthin isomers were not significantly different between plants cultivated at 3 mM and 12 mM, but significantly lower in plants cultivated at 0.75 mM compared to 12 mM (except for *all trans*-violaxanthin in green lettuce where the difference was not significant). Lactucaxanthin showed the same response in green lettuce. In red lettuce, however, there were significant differences between plants taken from all three nitrogen concentrations. With decreasing nitrogen availability, lactucaxanthin, in red lettuce, appears to be the first carotenoid negatively affected. Interestingly, also chlorophyll a concentration is already negatively affected at 3 compared to 12 mM N in red lettuce.

In some samples taken from the 0.75 mM N treatments, *all-trans* violaxanthin and lactucaxanthin were very close to or below the detection limit. These low concentrations offer an explanation for the large variation among the repeats, causing the relative differences regarding *all-trans* violaxanthin depicted in Fig 2 to be large but not significant.

Reports on a different physiological function of the two violaxanthin isomers *in planta* are scarce. Interestingly, *in vitro*, *all trans*-violaxanthin has been observed to convert into the *cis*-isomer as a result of illumination or heating, suggesting this mechanism to be an security valve consuming excessive excitation energy of the antenna pigments [29]. However, it’s not that simple: *Cis*-violaxanthin additionally acts as precursor for the plant hormone absisic acid [44]. This metabolic complexity may explain why the ratio between *all trans*- and *cis*-violaxanthin was not significantly influenced by the radiation or nitrogen treatments in our experiment.

The overall reduction of carotenoids does not correspond to the results obtained on algae [31]. Our findings agree with the general results on total carotenes published by Rouchaud et al. [34] obtained in a field experiment with butterhead lettuce. The single carotenoids’ response to the conditions studied here resembled each other.

In red lettuce, neither of the chlorophylls or carotenoids was significantly influenced by the radiation intensity (Table 3). In green lettuce however, shaded plants contained higher concentrations of chlorophyll b, lutein, lactucaxanthin and both violaxanthin isomers (Table 3). This suggests a greater physiological plasticity of green lettuce in response to radiation. No interaction between radiation intensity and nitrogen concentration was detected.

Carotenoids are part of the light harvesting complexes of the photosystems, involved in non-photochemical quenching and potent ROS scavengers [31]. In our experiment, their concentration decreased along with that of the chlorophylls, possibly to prevent any further absorption of radiation energy which could not be handled by the photosynthetic apparatus due to N limitations. In the 0.75 mM treatment, plants probably hardly accomplished any photosynthesis. This assumption is corroborated by the little head mass accumulated (see Table 1). Hughes et al. [45] suggested that, in red leaves, photoprotective anthocyanins might compensate for low capacities of non-photochemical quenching due to low concentrations of xanthophylls. Our results on red lettuce are in line with this hypothesis.

It is remarkable that the carotenoids did not respond like an antioxidant but rather as photosynthetic pigments. Proteins are crucial parts of the photosynthetic apparatus. All functional carotenoids in the thylakoid membrane are protein bound [28]. These complexes probably are a pivotal point of action for N limitation with detrimental effects on the photosynthetic apparatus. Phenolics and carotenoids differ in their physico-chemical qualities: Flavonoid glycosides and caffeic acid derivatives are water soluble while carotenoids are not. Hence, with different localization in the cell, their suitability to encounter oxidative challenges might depend on the origin and distribution of ROS.

### Sugars

We analyzed the concentration of glucose, fructose and sucrose and tested if they correlate to the concentration of the phenolic compounds. Sugar concentrations are listed in Table 4, correlation coefficients are listed in Table 5.

**Table 5:** Correlation coefficients for sugars and phenolic compounds in red and green lettuce.

		Cy3MG	Q3MG	Q3Gc/L7Gc	Chicoric acid	Chlorogenic acid
**Red Lettuce**	**Sucrose**	0.88 *	0.66 *	0.79 *	0.46	0.47 *
	**Glucose**	-0.79 *	-0.56 *	-0.71 *	-0.30	-0.31
	**Fructose**	-0.83 *	-0.61 *	-0.77 *	-0.39	-0.40
**Green Lettuce**	**Sucrose**		0.69 *	0.81 *	0.40	0.41
	**Glucose**		-0.61 *	-0.71 *	-0.45	-0.50 *
	**Fructose**		-0.72 *	-0.83 *	-0.53 *	-0.55 *

Concentrations of sugars and phenolic compounds are given in milligram per gram dry matter. Cy3MG = cyanidin-3-*O*-(6΄΄-*O*-malonyl)-glucoside, Q3MG = quercetin-3-*O*-(6΄΄-*O*-malonyl)-glucoside, Q3Gc/L7Gc = quercetin-3-*O*-glucuronide and luteolin-7-*O*-glucuronide. Green lettuce does not produce Cy3MG. Significant correlations are marked with an asterisk (α = 0.05).

The correlation analyses (Table 5) showed significant positive correlations between sucrose and all flavonoid glycosides in red and green lettuce as well as chlorogenic acid in red lettuce. This positive correlation is in line with previous reports in the literature, which on the one hand proposes an abundance of precursors for phenylpropanoid biosynthesis [24] to be the explanation or that sucrose acts as proximate trigger enhancing gene expression for enzymes involved in the biosynthesis [25]. The latter mechanism has only been demonstrated for anthocyanins.

Our analysis additionally showed significant negative correlations between glucose as well as fructose and all flavonoid glycosides in red and green lettuce (Table 5). In green lettuce, also chlorogenic acid was negatively correlated with glucose and fructose and chicoric acid with fructose. To our knowledge, similar results have not been reported before.

### Do our results support or contradict any existing theories regarding the underlying mechanisms of N deficiency?

Our results support the “photoprotection theory” originally proposed as an explanation for anthocyanin synthesis in autumn leaves and young leaves, as summarized by Archetti et al. [46]: In autumn, chlorophyll concentration decreases in leaves of deciduous trees due to nitrogen resorption whereas young leaves develop light capture ability first, before their CO_2_ assimilation is running at full capacity. N limited lettuce leaves in our experiment may have been in a very similar situation due to their low concentration of photosynthetic pigments and may have accumulated anthocyanins to protect a malfunctioning photosynthetic apparatus. Plants possess several mechanisms to detoxify reactive oxygen species, some of them are enzymatic like the superoxide dismutase, some non-enzymatic like phenolic compounds [15]. Gould and Lister [5] suggested that anthocyanins may be of greatest use to the plant in situations when other mechanisms are exhausted or impaired. During nitrogen deficiency, enzymatic detoxification may be impeded, creating the need for non-nitrogen-reliant alternatives. The malonylglucosides of quercetin and of cyanidin as well as chicoric acid extracted from lettuce are good absorbers of oxygen radicals [19].

**Fig 3:**
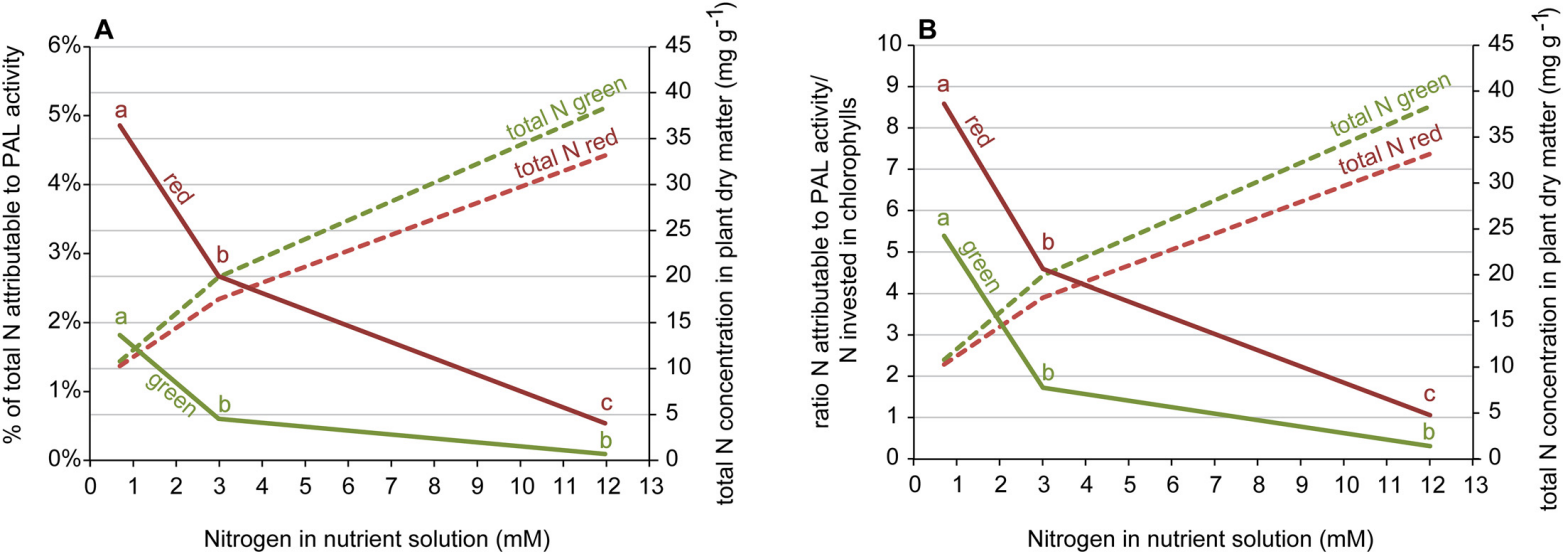
Calculations on the contribution of PAL activity to total nitrogen in the leaves and to nitrogen invested in chlorophyll, respectively. (A) Percentage of total nitrogen attributable to PAL activity and (B) the ratio between the amount of N attributable to PAL activity and the amount of N invested in chlorophyll a+b, each depicted together with the the total N concentration given in milligram per gram dry matter in red and green lettuce, cultivated with three different nitrogen concentrations in the nutrient solution, given in millimol. Identical letters at the graphs indicate that the means, evaluated per cultivar, do not differ significantly (two-way ANOVA, Tukey HSD test, α = 0.05, n = 3). N = nitrogen, PAL = phenylalanine ammonia-lyase.

Our results are furthermore in line with the “deamination theory” which suggests that increased concentration of ammonia through PAL activity drives the accumulation of phenolics. Not only one specific flavonoid was present in increased concentration but all detected caffeic acid derivatives and flavonoid glycosides (Table 2). The whole pathway appears to be enhanced. The percentage of total N attributable to PAL activity increased with decreasing nitrogen concentration in the nutrient solution, especially in red lettuce where it reached 4.9% in the 0.75 mM treatment (see Fig 3A and Table 2). The photosynthetic apparatus ranks very high on the list of priorities when it comes to distributing N: In pea leaves, up to 80% of leaf N can be allocated to chloroplasts and 20% of leaf N to the thylakoids alone [47]. Interestingly, in the 0.75 mM treatment, it was over eight times as much N as was fixed in chlorophyll while the percentage of N fixed in chlorophyll a+b was not influenced by the nitrogen concentration (Fig 3B and Table 2). Our calculations suggest that the amount of nitrogen supplied by PAL activity might actually make a real difference to plants experiencing severe nitrogen deficiency.

Our results largely support the “C/N ratio theory” which suggests that there is a general shift to carbon based metabolites under N deficiency. The response of flavonoids and caffeic acid derivatives response was in line. Also, looking at the big picture, a general shift towards carbon based compounds in nitrogen deficient plants is visible (see C/N ratio: Table 1 and sucrose concentration: Table 4). However, the metabolic regulation may be a bit more complex, not ignoring the compounds’ function: Like flavonoids, carotenoids are carbon based compounds that do not contain nitrogen. Yet they displayed a contrasting response to nitrogen deficiency. In the big picture, this does not show because even though their function is fundamentally linked to photosynthesis, their concentration is relatively low.

### Summary

In summary, our hypotheses 1–4 were not contradicted by our results and, hence, do not have to be discarded at this time: N deficiency resulted in increased concentration of flavonoid glycosides and caffeic acid derivatives in red and green lettuce. The concentration of chlorophyll a and b, β-carotene and xanthophylls decreased. Green lettuce showed a greater plasticity regarding its concentration of phenolic compounds and photosynthetic pigments than red lettuce. The concentrations of sugars correlated to those of the phenolics—positively in the case of sucrose, negatively regarding glucose and fructose. Little support for hypothesis 5 was detected. In contrast to our expectations, we only detected very few interaction between radiation intensity and nitrogen concentration.

Regarding underlying mechanisms of N depletion proposed in the literature, our results are generally in line with the “photoprotection thoery” and the “deamination theory”. Additionally, we found some support for the “C/N ratio theory”.

## Supporting Information

S1 Fig Diurnal variation of the radiation intensity inside the greenhouse. Depicted is the mean Photosynthetic photon flux density (PPFD) (of the previous hour) in μmol m^−2^ s^−1^, based on the last week before harvest.
(TIF)

S1 Table Results of 3-factorial ANOVA for phenolic compounds. Nitrogen (N) concentration in the nutrient solution is given in millimol per liter (mM). Photosynthetic photon flux density (PPFD) is given in μmol m-2 s-1. Phenolics concentration is given in milligram per gram dry matter. Data was evaluated via three-way ANOVA, factors: mM N, PPFD and genotype, α = 0.05, followed by Tukey HSD test (mean, n = 3). Identical letters indicate that values do not differ significantly. Asterisks indicate significantly influential factors. Cy3MG = cyanidin-3-O-(6΄΄-O-malonyl)-glucoside, Q3MG = quercetin-3-O-(6΄΄-O-malonyl)-glucoside, Q3Gc/L7Gc = quercetin-3-O-glucuronide and luteolin-7-O-glucuronide.
(DOC)

S2 Table Results of 3-factorial ANOVA for carotenoids. Nitrogen (N) concentration in the nutrient solution is given in millimol per liter (mM). Photosynthetic photon flux density (PPFD) is given in μmol m-2 s-1. Concentrations are given in milligram per gram dry matter. Data was evaluated via three-way ANOVA, factors: mM N, PPFD and genotype, α = 0.05, followed by Tukey HSD test (mean, n = 3). Identical letters indicate that values do not differ significantly. Asterisks indicate significantly influential factors.
(DOC)

S3 Table Results of 3-factorial ANOVA for growth characteristics, nitrogen, nitrate, carbon and sugar concentration. Nitrogen (N) concentration in the nutrient solution is given in millimol per liter (mM). Photosynthetic photon flux density (PPFD) is given in μmol m-2 s-1. Head mass is given in gram fresh matter. Nitrogen, carbon (C) and nitrate (NO3) concentration is given in milligram per gram dry matter. Data was evaluated via three-way ANOVA, factors: mM N, PPFD and genotype, α = 0,05, followed by Tukey HSD test (mean, n = 3). Identical letters indicate that values do not differ significantly. Asterisks indicate significantly influential factors.
(DOC)

## References

[1] Liu W, Zhu D-W, Liu D-H, Geng M-J, Zhou W-B, Mi WJ, et al (2010) Influence of nitrogen on the primary and secondary metabolism and synthesis of flavonoids in *Chrysanthemum morifolium* Ramat. Journal of Plant Nutrition 33: 240–254.

[2] Galloway JN, Townsend AR, Erisman JW, Bekunda M, Cai Z, Freney JR, et al (2008) Transformation of the nitrogen cycle: recent trends, questions, and potential solutions. Science 320: 889–892. 10.1126/science.1136674 18487183

[3] Treutter D (2010) Managing Phenol Contents in Crop Plants by Phytochemical Farming and Breeding-Visions and Constraints. International Journal of Molecular Sciences 11: 807–857. 10.3390/ijms11030807 20479987 PMC2868352

[4] Becker C, Klaering H-P, Kroh LW, Krumbein A (2013) Temporary reduction of radiation does not permanently reduce flavonoid glycosides and phenolic acids in red lettuce. Plant Physiology and Biochemistry 72: 154–160. 10.1016/j.plaphy.2013.05.006 23735845

[5] Gould KS, Lister C (2006) Flavonoid functions in plants In: Andersen Ø, Markham K, editors. Flavonoids: Chemistry, biochemistry and applications. pp. 397–441.

[6] Boudet AM (2007) Evolution and current status of research in phenolic compounds. Phytochemistry 68: 2722–2735. 17643453 10.1016/j.phytochem.2007.06.012

[7] Mulabagal V, Ngouajio M, Nair A, Zhang Y, Gottumukkala AL, Nair MG (2010) In Vitro evaluation of red and green lettuce (Lactuca sativa) for functional food properties. Food Chemistry 118: 300–306.

[8] Skibola CF, Smith MT (2000) Potential health impacts of excessive flavonoid intake. Free Radical Biology and Medicine 29: 375–383. 11035267 10.1016/s0891-5849(00)00304-x

[9] Stefanelli D, Winkler S, Jones R (2011) Reduced nitrogen availabililty during growth improves quality in red oak lettuce leaves by minimizing nitrate content, and increasing antioxidant capacity and leaf mineral content. Agricultural Sciences 2: 477–486.

[10] Becker C, Klaering H-P, Kroh LW, Krumbein A (2014) Cool-cultivated red leaf lettuce accumulates cyanidin-3-O-(6″-O-malonyl)-glucoside and caffeoylmalic acid. Food Chemistry 146: 404–411. 10.1016/j.foodchem.2013.09.061 24176360

[11] Rubio-Wilhelmi MdM, Sanchez-Rodriguez E, Leyva R, Blasco B, Romero L, Blumwald E, et al (2012) Response of carbon and nitrogen-rich metabolites to nitrogen deficiency in PSARK∷IPT tobacco plants. Plant Physiology and Biochemistry 57: 231–237. 10.1016/j.plaphy.2012.06.004 22738868

[12] Olsen KM, Slimestad R, Lea US, Brede C, Løvdal T, Ruoff P, et al (2009) Temperature and nitrogen effects on regulators and products of the flavonoid pathway: experimental and kinetic model studies. Plant, Cell & Environment 32: 286–299. 10.1111/j.1365-3040.2008.01920.x 19054348

[13] Larbat R, Olsen KM, Slimestad R, Løvdal T, Bénard C, Verheul M, et al (2012) Influence of repeated short-term nitrogen limitations on leaf phenolics metabolism in tomato. Phytochemistry 77: 119–128. 10.1016/j.phytochem.2012.02.004 22414312

[14] Løvdal T, Olsen KM, Slimestad R, Verheul M, Lillo C (2010) Synergetic effects of nitrogen depletion, temperature, and light on the content of phenolic compounds and gene expression in leaves of tomato. Phytochemistry 71: 605–613. 10.1016/j.phytochem.2009.12.014 20096428

[15] Gill SS, Tuteja N (2010) Reactive oxygen species and antioxidant machinery in abiotic stress tolerance in crop plants. Plant Physiology and Biochemistry 48: 909–930. 10.1016/j.plaphy.2010.08.016 20870416

[16] Konstantopoulou E, Kapotis G, Salachas G, Petropoulos SA, Chatzieustratiou E, Karapanos IC, et al (2012) Effect of nitrogen application on growth parameters, yield and leaf nitrate content of greenhouse lettuce cultivated during three seasons. Journal of Plant Nutrition 35: 1246–1254.

[17] Stewart AJ, Chapman W, Jenkins GI, Graham I, Martin T, Crozier A (2001) The effect of nitrogen and phosphorus deficiency on flavonol accumulation in plant tissues. Plant, Cell & Environment 24: 1189–1197.

[18] Rice-Evans C, Miller N, Paganga G (1997) Antioxidant properties of phenolic compounds. Trends in Plant Science 2: 152–159.

[19] Caldwell CR (2003) Alkylperoxyl radical scavenging activity of red leaf lettuce (Lactuca sativa L.) phenolics. Journal of agricultural and food chemistry 51: 4589–4595. 14705882 10.1021/jf030005q

[20] Steyn WJ, Wand SJE, Holcroft DM, Jacobs G (2002) Anthocyanins in vegetative tissues: a proposed unified function in photoprotection. New Phytologist 155: 349–361. 10.1046/j.1469-8137.2002.00482.x 33873306

[21] Lea US, Slimestad R, Smedvig P, Lillo C (2007) Nitrogen deficiency enhances expression of specific MYB and bHLH transcription factors and accumulation of end products in the flavonoid pathway. Planta 225: 1245–1253. 17053893 10.1007/s00425-006-0414-x

[22] Becker C, Klaering H-P, Schreiner M, Kroh LW, Krumbein A (2014) Unlike Quercetin Glycosides, Cyanidin Glycoside in Red Leaf Lettuce Responds More Sensitively to Increasing Low Radiation Intensity before than after Head Formation Has Started. Journal of Agricultural and Food Chemistry. 10.1021/jf404782n PMC4110108 24382136

[23] Zhang K, Li Z, Li Y, Li Y, Kong D, Wu RH (2013) Carbohydrate accumulation may be the proximate trigger of anthocyanin biosynthesis under autumn conditions in Begonia semperflorens. Plant Biology 15: 991–1000. 10.1111/j.1438-8677.2012.00721.x 23578316

[24] Ibrahim MH, Jaafar HZE, Rahmat A, Rahman ZA (2010) The Relationship between Phenolics and Flavonoids Production with Total Non Structural Carbohydrate and Photosynthetic Rate in Labisia pumila Benth. under High CO2 and Nitrogen Fertilization. Molecules 16: 162–174. 10.3390/molecules16010162 21191319 PMC6259453

[25] Teng S, Keurentjes J, Bentsink L, Koornneef M, Smeekens S (2005) Sucrose-specific induction of anthocyanin biosynthesis in Arabidopsis requires the MYB75/PAP1 gene. Plant Physiology 139: 1840–1852. 16299184 10.1104/pp.105.066688 PMC1310563

[26] Baslam M, Morales F, Garmendia I, Goicoechea N (2013) Nutritional quality of outer and inner leaves of green and red pigmented lettuces (Lactuca sativa L.) consumed as salads. Scientia Horticulturae 151: 103–111.

[27] Schopfer P, Brennicke A (2010) Pflanzenphysiologie: Spektrum Akademischer Verlag Heidelberg.

[28] Trebst A (2003) Function of beta-carotene and tocopherol in photosystem II. Zeitschrift fur Naturforschung C 58: 609–620. 10.1515/znc-2003-9-1001 14577617

[29] Niedz̀wiedzki D, Krupa Z, Gruszecki WI (2005) Temperature-induced isomerization of violaxanthin in organic solvents and in light-harvesting complex II. Journal of Photochemistry and Photobiology B: Biology 78: 109–114. 10.1016/j.jphotobiol.2004.09.012 15664497

[30] Phillip D, Young A (1995) Occurrence of the carotenoid lactucaxanthin in higher plant LHC II. Photosynthesis Research 43: 273–282. 10.1007/BF00029940 24306850

[31] Lamers PP, Janssen M, De Vos RC, Bino RJ, Wijffels RH (2008) Exploring and exploiting carotenoid accumulation in *Dunaliella salina* for cell-factory applications. Trends in biotechnology 26: 631–638. 10.1016/j.tibtech.2008.07.002 18752860

[32] Jahns P, Holzwarth AR (2012) The role of the xanthophyll cycle and of lutein in photoprotection of photosystem II. Biochimica et Biophysica Acta (BBA)—Bioenergetics 1817: 182–193. 21565154 10.1016/j.bbabio.2011.04.012

[33] Schachtman DP, Shin R (2007) Nutrient sensing and signaling: NPKS. Annual Review of Plant Biology 58: 47–69. 17067284 10.1146/annurev.arplant.58.032806.103750

[34] Rouchaud J, Moons C, Meyer JA, Benoit F, Ceustermans N, Linden F (1984) Effects of soil fertilization and covering of the culture with plastic film on the provitamin A content of early lettuces. Plant and Soil 80: 139–141.

[35] Coria-Cayupán YS, Sánchez de Pinto MI, Nazareno MA (2009) Variations in bioactive substance contents and crop yields of lettuce (Lactuca sativa L.) cultivated in soils with different fertilization treatments. Journal of Agricultural and Food Chemistry 57: 10122–10129. 10.1021/jf903019d 19821565

[36] Liaaen-Jensen S, Pfander H, Britton G (1995) Carotenoids Vol 1b: Spectroscopy: Birkhäuser.

[37] Klopotek Y, Kläring HP (2014) Accumulation and remobilisation of sugar and starch in the leaves of young tomato plants in response to temperature. Scientia Horticulturae 180: 262–267.

[38] Stamp N (2003) Out of the quagmire of plant defense hypotheses. The Quarterly Review of Biology 78: 23–55. 12661508 10.1086/367580

[39] Neilson EH, Goodger JQD, Woodrow IE, Møller BL (2013) Plant chemical defense: at what cost? Trends in Plant Science 18: 250–258. 10.1016/j.tplants.2013.01.001 23415056

[40] Fu WG, Li PP, Wu YY (2012) Effects of different light intensities on chlorophyll fluorescence characteristics and yield in lettuce. Scientia Horticulturae 135: 45–51.

[41] DuPont SM, Mondin Z, Williamson G, Price KR (2000) Effect of variety, processing and storage on the flavonoid glycoside content and composition of lettuce and endive. Journal of Agricultural and Food Chemistry 48: 3957–3964. 10995297 10.1021/jf0002387

[42] Tattini M, Landi M, Brunetti C, Giordano C, Remorini D, Gould KS, et al (2014) Epidermal coumaroyl anthocyanins protect sweet basil against excess light stress: multiple consequences of light attenuation. Physiologia Plantarum: n/a-n/a. 10.1111/ppl.12201 24684471

[43] Berges JA, Charlebois DO, Mauzerall DC, Falkowski PG (1996) Differential effects of nitrogen limitation on photosynthetic efficiency of photosystems I and II in microalgae. Plant Physiology 110: 689–696. 12226211 10.1104/pp.110.2.689 PMC157765

[44] Moise AR, Al-Babili S, Wurtzel ET (2013) Mechanistic Aspects of Carotenoid Biosynthesis. Chemical reviews 114: 164–193. 10.1021/cr400106y 24175570 PMC3898671

[45] Hughes NM, Burkey KO, Cavender-Bares J, Smith WK (2012) Xanthophyll cycle pigment and antioxidant profiles of winter-red (anthocyanic) and winter-green (acyanic) angiosperm evergreen species. Journal of Experimental Botany 63: 1895–1905. 10.1093/jxb/err362 22162871

[46] Archetti M, Döring TF, Hagen SB, Hughes NM, Leather SR, Lee DW, et al (2009) Unravelling the evolution of autumn colours: an interdisciplinary approach. Trends in ecology & evolution 24: 166–173. 19178979 10.1016/j.tree.2008.10.006

[47] Makino A, Osmond B (1991) Effects of nitrogen nutrition on nitrogen partitioning between chloroplasts and mitochondria in pea and wheat. Plant Physiology 96: 355–362. 16668193 10.1104/pp.96.2.355 PMC1080777

