# Supplementary figures and images for: Nitrogen Limited Red and Green Leaf Lettuce Accumulate Flavonoid Glycosides, Caffeic Acid Derivatives, and Sucrose while Losing Chlorophylls, Β-Carotene and Xanthophylls
Source: PLoS One. 2015 Nov 16;10(11):e0142867. doi: 10.1371/journal.pone.0142867 (PMC4646504; doi:10.1371/journal.pone.0142867)

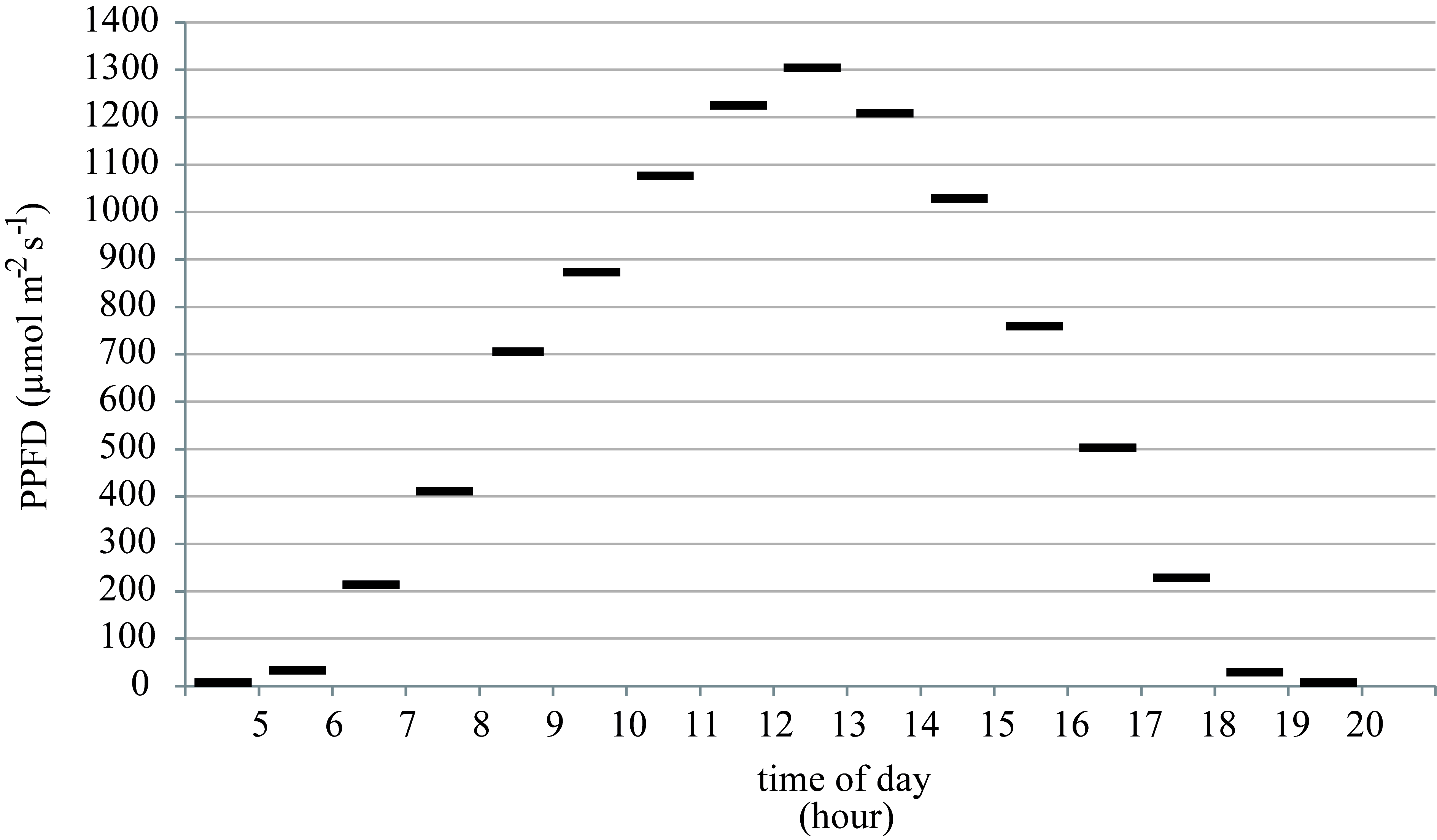

Supplement: S1 Fig — Depicted is the mean Photosynthetic photon flux density (PPFD) (of the previous hour) in μmol m−2 s−1, based on the last week before harvest. (TIF) [file pone.0142867.s001.tif]
