# Supplementary material for: Nitrogen Limited Red and Green Leaf Lettuce Accumulate Flavonoid Glycosides, Caffeic Acid Derivatives, and Sucrose while Losing Chlorophylls, Β-Carotene and Xanthophylls
Source: PLoS One. 2015 Nov 16;10(11):e0142867. doi: 10.1371/journal.pone.0142867 (PMC4646504; doi:10.1371/journal.pone.0142867)
Supplement: S1 Table — Nitrogen (N) concentration in the nutrient solution is given in millimol per liter (mM). Photosynthetic photon flux density (PPFD) is given in μmol m-2 s-1. Phenolics concentration is given in milligram per gram dry matter. Data was evaluated via three-way ANOVA, factors: mM N, PPFD and genotype, α = 0.05, followed by Tukey HSD test (mean, n = 3). Identical letters indicate that values do not differ significantly. Asterisks indicate significantly influential factors. Cy3MG = cyanidin-3-O-(6΄΄-O-malonyl)-glucoside, Q3MG = quercetin-3-O-(6΄΄-O-malonyl)-glucoside, Q3Gc/L7Gc = quercetin-3-O-glucuronide and luteolin-7-O-glucuronide. (DOC) [file pone.0142867.s002.doc]

S1 Table: Results of 3-factorial ANOVA for phenolic compounds.

|  |  | **Q3Gc/L7Gc** | **Q3MG** | **Chicoric acid** | **Chlorogenic acid** |
| --- | --- | --- | --- | --- | --- |
| **Main effects** |  |  |  |  |  |
| N | 0.75 | 1.57 a | 3.85 a | 2.24 a | 0.82 a |
|  | 3 | 1.07 b | 3.25 a | 2.16 a | 0.77 a |
|  | 12 | 0.45 c | 1.21 b | 0.72 b | 0.29 b |
|  |  |  |  |  |  |
| PPFD | 678 µmol | 1.12 a | 3.04 a | 1.69 | 0.62 |
|  | 339 µmol | 0.94 b | 2.50 b | 1.72 | 0.63 |
|  |  |  |  |  |  |
| genotype | red | 1.38 a | 4.40 a | 2.48 a | 1.00 a |
|  | green | 0.68 b | 1.13 b | 0.93 b | 0.25 b |
|  |  |  |  |  |  |
| **Significance** | N | ***** | ***** | ***** | ***** |
|  | PPFD | ***** | ***** | ns | ns |
|  | N * PPFD | ns | ns | ns | ns |
|  | genotype | ***** | ***** | ***** | ***** |
|  | N* genotype | ns (p=0.05) | ***** | ***** | ***** |

Nitrogen (N) concentration in the nutrient solution is given in millimol per liter (mM). Photosynthetic photon flux density (PPFD) is given in µmol m^-2^ s^-1^. Phenolics concentration is given in milligram per gram dry matter. Data was evaluated via three-way ANOVA, factors: mM N, PPFD and genotype, α=0.05, followed by Tukey HSD test (mean, n = 3). Identical letters indicate that values do not differ significantly. Asterisks indicate significantly influential factors. Cy3MG = cyanidin-3-O-(6΄΄-O-malonyl)-glucoside, Q3MG = quercetin-3-O-(6΄΄-O-malonyl)-glucoside, Q3Gc/L7Gc = quercetin-3-O-glucuronide and luteolin-7-O-glucuronide.
