# Supplementary material for: Nitrogen Limited Red and Green Leaf Lettuce Accumulate Flavonoid Glycosides, Caffeic Acid Derivatives, and Sucrose while Losing Chlorophylls, Β-Carotene and Xanthophylls
Source: PLoS One. 2015 Nov 16;10(11):e0142867. doi: 10.1371/journal.pone.0142867 (PMC4646504; doi:10.1371/journal.pone.0142867)
Supplement: S2 Table — Nitrogen (N) concentration in the nutrient solution is given in millimol per liter (mM). Photosynthetic photon flux density (PPFD) is given in μmol m-2 s-1. Concentrations are given in milligram per gram dry matter. Data was evaluated via three-way ANOVA, factors: mM N, PPFD and genotype, α = 0.05, followed by Tukey HSD test (mean, n = 3). Identical letters indicate that values do not differ significantly. Asterisks indicate significantly influential factors. (DOC) [file pone.0142867.s003.doc]

S2 Table: Results of 3-factorial ANOVA for carotenoids and chlorophylls.

| **Main effects** |  | **Chlorophyll a** | **Chlorophyll b** | **β-Carotene** | **Lutein** | **Lactucaxanthin** | **Neoxanthin** | ***all trans-V*iolaxanthin** | ***cis-V*iolaxanthin** |
| --- | --- | --- | --- | --- | --- | --- | --- | --- | --- |
| N | 0.75 | 0.49 c | 0.35 c | 0.048 b | 0.07 | 0.008 c | 0.035 b | 0.0003 b | 0.016 c |
|  | 3 | 0.91 b | 0.68 b | 0.091 a | 0.12 | 0.060 b | 0.130 a | 0.0012 ab | 0.037 b |
|  | 12 | 1.64 a | 0.92 a | 0.089 a | 0.13 | 0.090 a | 0.138 a | 0.0028 a | 0.048 a |
|  |  |  |  |  |  |  |  |  |  |
| PPFD | 678 µmol | 0.94 | 0.59 b | 0.07 | 0.10 b | 0.042 b | 0.086 b | 0.0010 | 0.029 b |
|  | 339 µmol | 1.09 | 0.71 a | 0.08 | 0.12 a | 0.065 a | 0.116 a | 0.0019 | 0.038 a |
|  |  |  |  |  |  |  |  |  |  |
| genotype | red | 1.06 | 0.73 a | 0.088 a | 0.12 a | 0.067 a | 0.124 a | 0.0018 | 0.040 a |
|  | green | 0.96 | 0.57 b | 0.064 b | 0.09 b | 0.039 b | 0.078 b | 0.0011 | 0.027 b |
|  |  |  |  |  |  |  |  |  |  |
| **Significance** | N | * | * | * | * | * | * | * | * |
|  | PPFD | ns | * | ns | * | * | * | ns | * |
|  | N * PPFD | ns | ns | ns | ns | ns | ns | ns | ns |
|  | genotype | ns | * | * | * | * | * | ns | * |
|  | N* genotype | ns | ns | ns | ns | ns | ns | ns | ns |

Nitrogen (N) concentration in the nutrient solution is given in millimol per liter (mM). Photosynthetic photon flux density (PPFD) is given in µmol m^-2^ s^-1^. Concentrations are given in milligram per gram dry matter. Data was evaluated via three-way ANOVA, factors: mM N, PPFD and genotype, α=0.05, followed by Tukey HSD test (mean, n = 3). Identical letters indicate that values do not differ significantly. Asterisks indicate significantly influential factors.
