# Supplementary material for: Nitrogen Limited Red and Green Leaf Lettuce Accumulate Flavonoid Glycosides, Caffeic Acid Derivatives, and Sucrose while Losing Chlorophylls, Β-Carotene and Xanthophylls
Source: PLoS One. 2015 Nov 16;10(11):e0142867. doi: 10.1371/journal.pone.0142867 (PMC4646504; doi:10.1371/journal.pone.0142867)
Supplement: S3 Table — Nitrogen (N) concentration in the nutrient solution is given in millimol per liter (mM). Photosynthetic photon flux density (PPFD) is given in μmol m-2 s-1. Head mass is given in gram fresh matter. Nitrogen, carbon (C) and nitrate (NO3) concentration is given in milligram per gram dry matter. Data was evaluated via three-way ANOVA, factors: mM N, PPFD and genotype, α = 0,05, followed by Tukey HSD test (mean, n = 3). Identical letters indicate that values do not differ significantly. Asterisks indicate significantly influential factors. (DOC) [file pone.0142867.s004.doc]

S3 Table: Results of 3-factorial ANOVA for growth characteristics, nitrogen, nitrate, carbon and sugar concentration.

| **Main effects** |  | **Head mass** | **number of leaves** | **total N** | **NO_3_** | **total C** | **glucose** | **fructose** | **sucrose** |
| --- | --- | --- | --- | --- | --- | --- | --- | --- | --- |
| N | 0.75 | 22.68 c | 12.42 c | 10.54 c | 0.20 b | 396.11 a | 29.51 b | 31.49 b | 191.32 a |
|  | 3 | 84.95 b | 19.58 b | 18.74 b | 0.20 b | 378.72 b | 75.92 a | 99.50 a | 69.50 b |
|  | 12 | 235.57 a | 25.00 a | 35.76 a | 7.26 a | 375.47 c | 71.77 a | 106.04 a | 39.74 a |
|  |  |  |  |  |  |  |  |  |  |
| PPFD | 678 µmol | 118.96 | 19.06 | 20.86 b | 2.37 b | 385.75 a | 56.88 | 77.50 | 106.81 |
|  | 339 µmol | 116.46 | 19.26 | 22.49 a | 2.73 a | 381.12 b | 61.26 | 80.52 | 93.57 |
|  |  |  |  |  |  |  |  |  |  |
| genotype | red | 99.88 b | 17.26 b | 20.33 b | 1.78 b | 387.93 a | 58.68 | 81.71 | 104.02 |
|  | green | 136.46 a | 21.17 a | 23.03 a | 3.32 a | 378.93 b | 59.45 | 76.31 | 96.35 |
|  |  |  |  |  |  |  |  |  |  |
| **Significance** | N | * | * | * | * | * | * | * | * |
|  | PPFD | ns | ns | * | * | * | ns | ns | ns |
|  | N * PPFD | ns | ns | ns | * | * | ns | ns | ns |
|  | genotype | * | * | * | * | * | ns | ns | ns |
|  | N* genotype | * | ns | * | * | * | ns | ns | * |

Nitrogen (N) concentration in the nutrient solution is given in millimol per liter (mM). Photosynthetic photon flux density (PPFD) is given in µmol m^-2^ s^-1^. Head mass is given in gram fresh matter. Nitrogen, carbon (C) and nitrate (NO_3_) concentration is given in milligram per gram dry matter. Data was evaluated via three-way ANOVA, factors: mM N, PPFD and genotype, α=0,05, followed by Tukey HSD test (mean, n = 3). Identical letters indicate that values do not differ significantly. Asterisks indicate significantly influential factors.
